# Intravenous tirofiban in acute ischemic stroke patients not receiving reperfusion treatments: a systematic review and meta-analysis of randomized controlled trials

**DOI:** 10.3389/fneur.2025.1552658

**Published:** 2025-05-13

**Authors:** Shatha Alqurashi, Mohammed S Alqahtani, Shahad Mohammed Albeladi, Saleha Almahdawi, Hala Danish, Hatoon Alshaikh, Ahmed Alkhiri, Ammar Alkawi, Fahad S Al-Ajlan, Adel Alhazzani

**Affiliations:** ^1^College of Medicine, King Saud Bin Abdulaziz University for Health Sciences, Jeddah, Saudi Arabia; ^2^King Abdullah International Medical Research Center, Jeddah, Saudi Arabia; ^3^Neuroscience Center, King Faisal Specialist Hospital and Research Centre, Riyadh, Saudi Arabia; ^4^Armed Forces Hospital, Southern Region, Khamis Mushait, Saudi Arabia; ^5^College of Medicine, Alfaisal University, Riyadh, Saudi Arabia

**Keywords:** stroke, tirofiban, systematic review, meta-analysis, ischemic stroke

## Abstract

**Background:**

Reperfusion treatments with intravenous thrombolysis and endovascular thrombectomy after acute ischemic stroke (AIS) can improve patients’ outcomes significantly. Yet, a substantial portion of patients miss the opportunity to receive reperfusion treatments. In this study, we aimed to assess the role of intravenous tirofiban in this specific population.

**Methods:**

A search was performed in Embase, Cochrane Central Register of Controlled Trials, Medline, and Web of Science databases from inception until August 2024. The random-effects model was used to calculate odds ratios (ORs) with their corresponding 95% confidence intervals (CIs). Efficacy endpoints included excellent (modified Rankin scale of 0–1) and good (modified Rankin scale of 0–2) functional outcomes at 90 days. Safety outcomes included symptomatic intracerebral hemorrhage (sICH), any ICH, and 90-day mortality.

**Results:**

Four randomized clinical trials, including a total of 1,199 patients, were included. Of these, 599 patients (50%) received tirofiban. The meta-analysis demonstrated that tirofiban was associated with significantly higher rates of both excellent (OR 1.63 [95% CI, 1.24–2.13]; I^2^ = 0) and good (OR 1.65 [95% CI, 1.19–2.29]; I^2^ = 0) functional outcomes at 90 days. No significant differences were observed in sICH, any ICH, or 90-days mortality.

**Conclusion:**

Treatment with intravenous tirofiban can be beneficial without increased risk in patients with AIS who are not eligible for reperfusion treatment. Further studies are still needed to validate the generalizability of these findings.

**Systematic review registration:**

https://www.crd.york.ac.uk/PROSPERO/view/CRD42024590097, CRD42024590097.

## 1 Introduction

Patients with acute ischemic stroke (AIS) are at increased risk of disability and mortality (1). Reperfusion treatments including intravenous thrombolysis and endovascular thrombectomy have established role and can improve stroke outcomes (2). However, the narrow therapeutic potential including short treatment window, risk of hemorrhagic transformation, and contraindications represent substantial challenges that limit their use in many cases (3). In such scenarios, the primary treatment option in most cases is oral antithrombotic agents. Factors like the presence of dysphagia and the risk of aspiration can limit or delay the administration of oral medications (2).

Tirofiban functions by reversibly inhibiting glycoprotein GP IIb/IIla, thereby inhibiting platelet aggregation and halting thrombosis (4). Tirofiban is widely used in patients with acute coronary syndrome, where its early administration can lower the risk of vascular complications and decrease the need for revascularization (5–7). Previous studies have attempted to assess the role of tirofiban in patients with AIS receiving IVT or EVT (8, 9). However, there is a lack of evidence regarding its safety and efficacy in patients with AIS who are ineligible for reperfusion therapies. Therefore, this systematic review and meta-analysis of randomized clinical trials (RCTs) aims to assess the safety and efficacy of tirofiban in patients with AIS who did not receive reperfusion treatments.

## 2 Methods

This systematic review and meta-analysis was carried out in accordance with the Preferred Reporting Items for Systematic Reviews and Meta-Analyses (PRISMA) (10). Ethical approvals and patients consents were not required as this study involves an analysis of aggregated data from prior published studies. The review followed a prespecified protocol registered with PROSPERO (CRD42024590097).

### 2.1 Search strategy

From inception until August 23, 2024, a systematic search was performed across Embase, Cochrane Central Register of Controlled Trials (CENTRAL), PubMed/Medline, and Web of Science databases. The search algorithm contained combinations of keywords related to acute ischemic stroke and tirofiban tailored to each database. A detailed search strategy is shown in Supplementary Table S1.

### 2.2 Study selection

Two independent investigators initially screened titles and abstracts, followed by a detailed full-text assessment. This review specifically examined RCTs that compared the safety and efficacy of tirofiban with other antiplatelet agents in patients with AIS. Excluded from our analysis were trials involving participants receiving IVT or EVT, studies comparing tirofiban with non-antiplatelet agents, observational studies, single-arm studies, conference papers, case reports, non-English studies, and review articles.

### 2.3 Data extraction and quality assessment

Using predetermined data extraction forms, two independent authors collected data related to study characteristics, patients baseline details, detailed treatment regimens, and outcomes of interest. To assess the risk of bias of RCTS, Cochrane’s Risk of Bias Tool 2 (RoB2) was used (11). Any disagreements were addressed through consensus with a third author.

### 2.4 Outcome measures

Efficacy endpoints included the excellent functional outcome, defined as a modified Rankin Scale (mRS) score of 0–1 at 90 days, and the good functional outcome, defined as a modified Rankin Scale (mRS) score of 0–2 at 90 days. Safety endpoints included rates of symptomatic intracerebral hemorrhage (sICH) as defined by each study, any ICH, and 90-day mortality.

### 2.5 Data analysis

RevMan software was used for data analysis. The inverse variance method with the random-effects model were used to pool endpoint data. A forest plot was created for each outcome. Statistical significance was established at a *p*-value < 0.05. Odds ratios (OR) with their corresponding 95% confidence intervals (CIs) were calculated to assess dichotomous variables. Cochran’s Q test and the Higgins I^2^ statistic were used to evaluate heterogeneity among studies, where *p* < 0.05 or I^2^ exceeding 50% were regarded as significant heterogeneity. Publication bias could not be evaluated using Egger’s test because fewer than 10 studies were included in this study (12).

## 3 Results

### 3.1 Study selection

**Figure 1:**
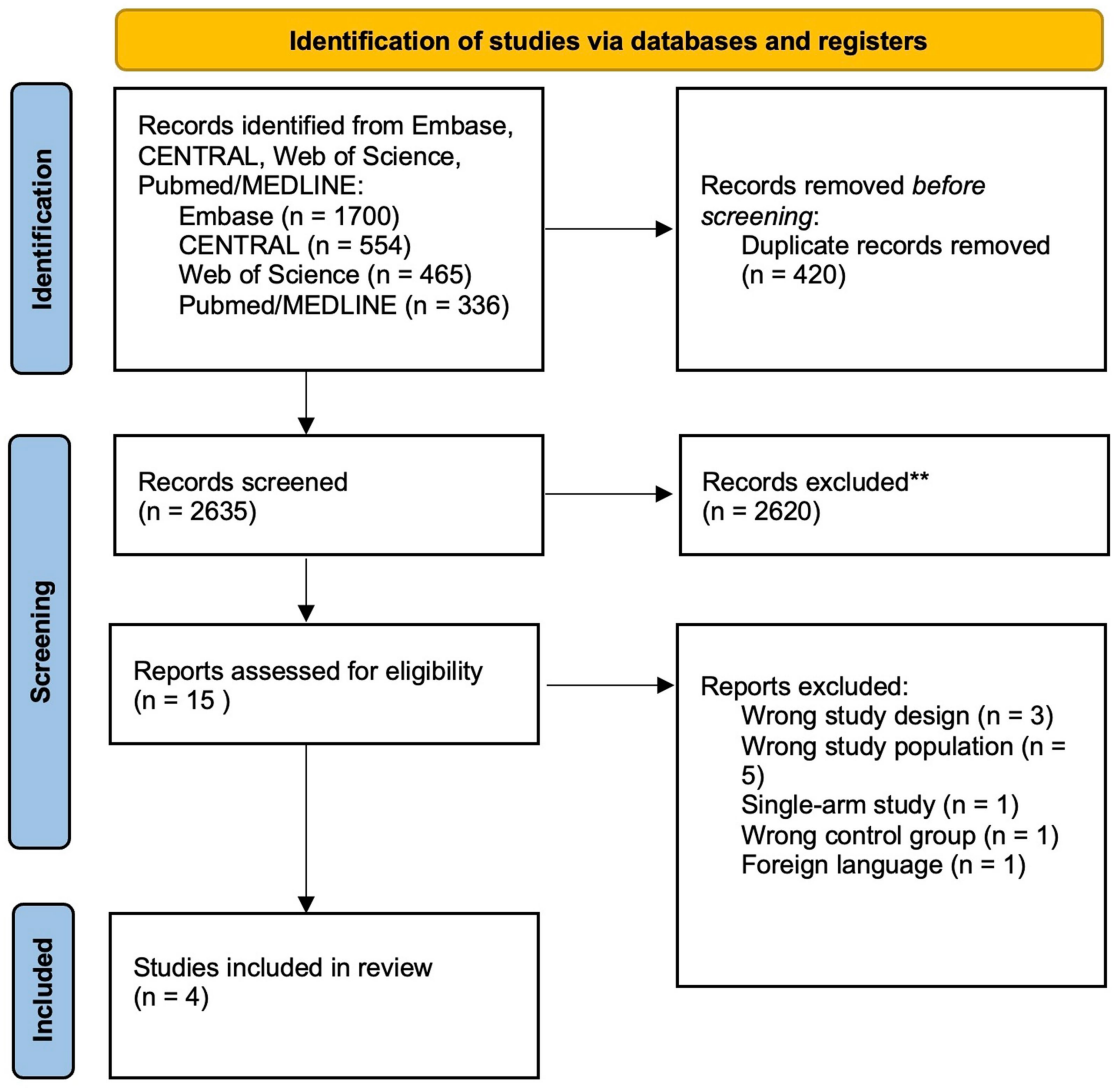
Preferred reporting items for systematic reviews and meta-analysis (PRISMA) flow diagram.

A total of 3,055 records had been exported from the included databases. These records were screened for duplicates and investigated for eligibility. A screening by abstract and title was done, in which 420 articles were duplicates, and 2,620 articles were excluded. The remaining 15 articles underwent a full-text assessment. Finally, four RCTs were included (13–16). Further details on the study selection process are illustrated in Figure 1.

### 3.2 Study characteristics

A total of 1,199 participants were included. Of those, 599 (50%) patients were in the tirofiban group and 600 (50%) patients in the control group. All included studies compared tirofiban to antiplatelet agents, which was either aspirin or/and clopidogrel. In all included RCTs, tirofiban was administered intravenously. Three RCTs have been conducted in China (14–16), while one RCTs was conducted in Italy (13). Among all included patients, there were 779 (65%) males and 420 (35%) females. For further details, refer to study summaries in (Table 1), patient baseline characteristics in (Table 2), and intervention details in (Table 3).

**Table 1:** Study characteristics.

Study ID	Country	Duration	Study design	Sample size		Main inclusion criteria	Main exclusion criteria	sICH definitions
				Tirofiban	Control			
Torgano et al. (13), 2010	Italy	2003–2006	Multicenter RCT	75	75	20–90 years old NIHSS score of 5–25 Symptom duration 160 min Exclusion of hemorrhage on brain CT Onset of stroke ≤ 6 h	ICH Seizure at onset of stroke Uncontrolled severe hypertension.	ECASS I
Han et al. (14), 2022	China	2020–2021	Multicenter RCT	177	180	≥ 18 years old Mild-to-moderate stroke (NIHSS score, 4–15) Within 12 h after stroke onset	Undergoing IVT or EVT treatment mRS score of ≥ 2 Cardioembolic stroke Atrial fibrillation	Heidelberg Bleeding Classification
Yu et al. (15), 2022	China	NR	Single-center RCT	134	133	18–85 years old Within 72 h of stroke onset from admission Without arterial occlusion or large area of hypoperfusion NIHSS score ≤ 20 on admission	LVO requiring EVT IVT treatment ICH pre-stroke mRS ≥ 2	ICH on follow-up head CT scan causing a 4 points decrease on NIHSS score
Zhao et al. (16), 2024	China	2020–2023	Multicenter RCT	213	212	18–80 years old NIHSS 4–20 Within 24 h of symptom onset or time last known well Motor examination rating scale of ≥ 2	IVT or EVT treatment Cardioembolic stroke or AIS due to other causes Pre-stroke mRS ≥ 2	ECASS III

sICH, symptomatic hemorrhage; RCT, randomized clinical trials; AIS, acute ischemic stroke; LVO, large vessel occlusion; EVT, endovascular therapy; IVT, intravenous thrombolysis; NIHSS, National Institutes of Health Stroke Scale; mRS, modified rankin score; ICH, intracerebral hemorrhage; CT, computed tomography; ECASS, European Cooperative Acute Stroke Study; NR, no records.

**Table 2:** Baseline patients’ characteristics.

Study ID	Male, *N* (%)		Age, median (range)		Admission NIHSS, median (range)		TOAST classification, *N* (%)				Risk factors, *N* (%)					
	Control	Tirofiban	Control	Tirofiban	Control	Tirofiban	Large artery atherosclerosis	Small vessel occlusion	Cardio-embolism	Other	HTN	DM	Dyslipidemia	CAD	Previous stroke/TIA	Smoking
Torgano et al. (13), 2010	37 (49.3)	36 (48)	73.8 (8.9)^a^	71.8 (13.7)^a^	9 (7–14)	9 (6–16)	AP: 20 (27) Tirofiban: 18 (24)	AP: 10 (13) Tirofiban: 15 (20)	AP: 27 (36) Tirofiban: 20 (27)	Undetermined AP: 18 (24) Tirofiban: 22 (29)	AP: 59 (79) Tirofiban: 49 (65)	AP: 17 (22) Tirofiban: 8 (11)	AP: 31 (41) Tirofiban: 27 (36)	NR	AP: 19 (25) Tirofiban: 10 (13)	AP: 20 (27) Tirofiban: 13 (17)
Han et al. (14), 2022	126 (70)	115 (65)	67 (59–75)	67 (59–74)	NR	NR	AP: 86 (47.8) Tirofiban: 72 (40.7)	AP: 94 (52.2) Tirofiban: 105 (59.3)	-	-	AP: 130 (72.2) Tirofiban: 120 (67.8)	AP: 52 (28.9) Tirofiban: 56 (31.6)	NR	AP: 59 (32.8) Tirofiban: 49 (27.7)	AP: 39 (21.7) Tirofiban: 38 (21.5)	NR
Yu et al. (15), 2022	78 (58.6)	86 (64.2)	71 (42–85)	68 (38–85)	6 (3–20)	5 (3–19)	AP: 55 (41.4) Tirofiban: 49 (36.6)	AP: 72 (54.1) Tirofiban: 73 (54.5)	AP: 6 (4.51) Tirofiban: 12 (8.9)	-	AP: 81 (60.9) Tirofiban: 91 (67.9)	AP: 36 (27) Tirofiban: 46 (34.4)	NR	AP: 22 (16.5) Tirofiban: 15 (11.2)	AP: 45 (33.8) Tirofiban: 46 (34.3)	AP: 46 (34.6) Tirofiban: 54 (40.3)
Zhao et al. (16), 2024	147 (69.3)	154 (72.3)	64 (56–71)	64 (56–70)	5 (4–8)	5 (4–7)	AP: 65 (30.7) Tirofiban: 49 (23.0)	AP: 52 (24.5) Tirofiban: 72 (33.8)	AP: 5 (2.4) Tirofiban: 4 (1.9)	Determined: AP: 3 (1.4) Tirofiban: 0 Undetermined: AP: 87 (41) Tirofiban: 88 (41.3)	AP: 134 (63.2) Tirofiban: 132 (62.0)	AP: 67 (31.6) Tirofiban: 68 (31.9)	AP: 25 (11.8) Tirofiban: 24 (11.3)	AP: 28 (13.2) Tirofiban: 27 (12.7)	AP: 66 (31.1) Tirofiban: 62 (29.1)	Previous smoking: AP: 103 (48.6) Tirofiban: 97 (45.5) Current smoking: AP: 87 (41) Tirofiban: 82 (38.5)

NIHSS, National Institutes of Health Stroke Scale; TOAST, Trial Org 10,172 in Acute Stroke Treatment; HTN, hypertension; DM, diabetes mellitus; CAD, coronary artery disease; TIA, transient ischemic attack; NR, no record. ^a^Data presented as mean (SD).

**Table 3:** Treatment protocols.

Study ID	Tirofiban route of administration	Tirofiban dose and time window	Oral AP protocol in Tirofiban group	Control group treatment protocol	Onset to treatment time median (range)
Torgano et al. (13), 2010	IV	0.6 μg/kg/min (30 min) +0.15 μg/kg/min (72 hours) Within 6 h	NR	IV, 300 mg aspirin for 3 days	AP: 4.4 (1.13)^a^ Tirofiban: 4.4 (1.06)^a^
Han et al. (14), 2022	IV	0.4 μg/kg/min (30 min) +0.1 μg/kg/min (48 h) Within 12 h	4 h before the end of the tirofiban treatment, 100 mg aspirin was given for 90 days	100 mg of aspirin for 90 days	NR
Yu et al. (15), 2022	IV	0.4 μg/kg/min (30 min) +0.1 μg/kg/min (72–108 h) Within 72 h	4 h before the end of the tirofiban treatment, 100 mg of oral aspirin and/or 75 mg of clopidogrel were given for 90 days	300 mg loading dose then 100 mg of aspirin and/or 300 mg loading dose then 75 mg of clopidogrel	AP: 12 (1–72) Tirofiban: 7.25 (0.5–72)
Zhao et al. (16), 2024	IV	0.4 μg/kg/min (30 min) +0.1 μg/kg/min (71.5 h) Within 24 h	4 h before the end of the tirofiban treatment, 150–300 mg/d of aspirin was given during the first 2 weeks then 100–300 mg/d after that for secondary prevention.	150–300 mg/d of aspirin during the first 2 weeks then 100–300 mg/d after that for secondary prevention	AP: 10.5 (6.6–21) Tirofiban: 12.5 (7.8–19.2)

AP, antiplatelets; IV, intravenous; IA, intraarterial; NR, no records. ^a^Data presented as mean (SD).

### 3.3 Risk of bias

Details of the risk of bias assessments can be found in Supplementary material. In summary, the four RCTs consistently demonstrated low bias risk across all assessed domains (Supplementary Table S2) (13–16).

### 3.4 Quantitative data synthesis

#### 3.4.1 Excellent functional outcome (mRS 0–1 at 90 days)

**Figure 2:**
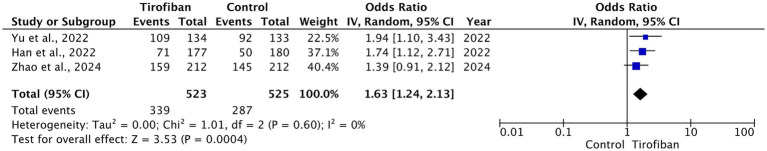
Forest plot and meta-analysis of excellent functional outcomes (mRS 0–1) at 90 days.

Three RCTs were included in the analysis. The pooled analysis demonstrated that tirofiban was significantly associated with higher rates of excellent functional outcomes with homogeneous effect (OR = 1.63, 95% CI [1.24, 2.13], *p* = 0.0004, I^2^ = 0%) (Figure 2) (14–16).

#### 3.4.2 Good functional outcome (mRS 0–2 at 90 days)

**Figure 3:**
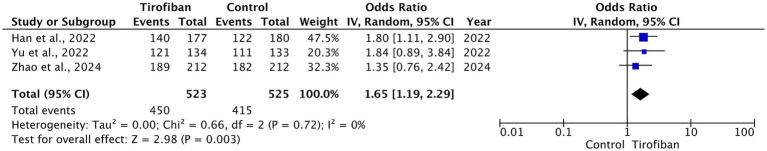
Forest plot and meta-analysis of good functional outcomes (mRS 0–2) at 90 days.

Three RCTs were included in this analysis. Significantly higher rates of good functional outcome were observed in the tirofiban group compared to control with homogeneous effect in the pooled analysis (OR = 1.65, 95% CI [1.19, 2.29], *p* = 0.003, I^2^ = 0%) (Figure 3) (14–16).

#### 3.4.3 sICH

**Figure 4:**
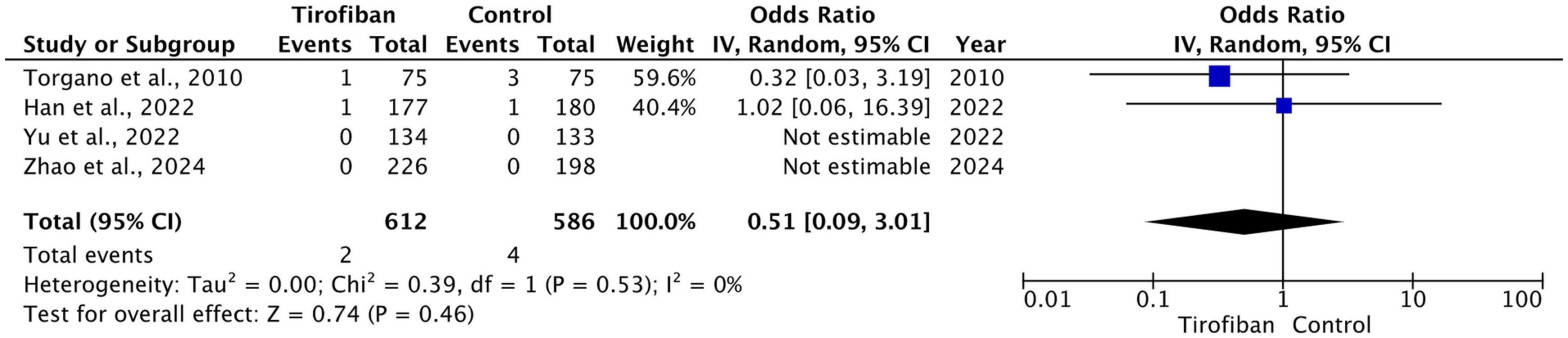
Forest plot and meta-analysis of sICH.

The total incidence of sICH was 6 (0.5%), of which 2 (0.3%) pertain to the tirofiban group and 4 (0.7%) pertain to the control groups. All included RCTs reported on sICH rates with no significant differences between the two groups (OR = 0.51, 95% CI [0.09, 3.01], *p* = 0.46, I^2^ = 0%) (Figure 4) (13–16).

#### 3.4.4 Any ICH

**Figure 5:**
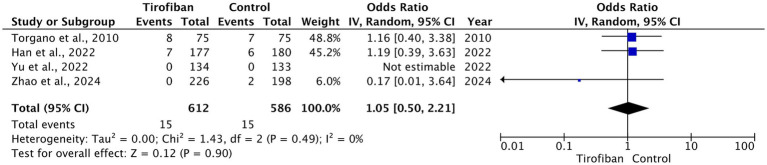
Forest plot and meta-analysis of any ICH.

The overall occurrence of ICH was 30 (2.5%), which includes 15 (2.5%) related to the tirofiban group and 15 (2.6%) to the control group. Pooled analysis from all RCTs demonstrated no statistically significant difference between the two groups with homogeneous effect (OR = 1.05, 95% CI [0.50, 2.21], *p* = 0.90, I^2^ = 0%) (Figure 5) (13–16).

#### 3.4.5 90-day mortality

**Figure 6:**
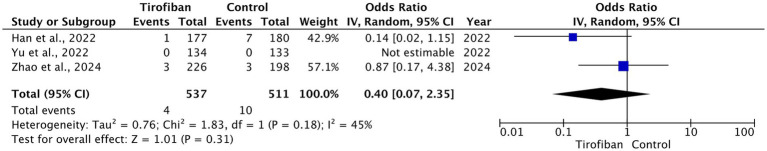
Forest plot and meta-analysis of 90-day mortality.

The overall 90-day mortality rate was 14 (1.3%). Four (0.7%) cases occurred in tirofiban group, while 10 (2%) cases occurred in the control group. Pooled analysis of three RCTs showed no statistically significant difference between the tirofiban and control groups with moderate heterogeneity (OR = 0.40, 95% CI [0.07, 2.35], *p* = 0.31, I^2^ = 45%) (Figure 6) (14–16).

## 4 Discussion

This systematic review and meta-analysis aimed to evaluate the safety and efficacy of tirofiban in patients with AIS who did not receive any reperfusion therapy. Results showed that tirofiban was associated with significantly higher rates of excellent and good functional outcomes after 90 days. In terms of safety outcomes, IV tirofiban was safe without increased rates of sICH, any ICH, or mortality.

Although IVT and EVT have been extensively studied and approved for patients with AIS, they are critically time-dependent, and patients must meet specific criteria to receive these treatments (2, 17–19). Prompt treatment administration is critical in the management of stroke. Nevertheless, pre-hospital and in-hospital delays represent a significant challenge in the timely management of acute stroke, where only around one-third of cases are seen within the critical time frame for the treatment with IVT or EVT, and less than 7% actually receive these treatments (17–20). For patients with AIS who are ineligible for intravenous or/and endovascular reperfusion therapy, the only treatment option available is oral antiplatelet treatment (2). This accentuates the importance of further research evaluating alternative treatment options for such a patient population. Tirofiban is a fast-acting antiplatelet agent that inhibits the glycoprotein IIb/IIIa receptors on platelets and can be administered intra-arterially or intravenously (21). In the early phase of stroke management, it is essential for patients to achieve a rapid and effective antiplatelet effect. Compared to the delayed onset of action of oral antiplatelet agents, tirofiban serves as a rapid-acting antiplatelet medication, typically within minutes post-injection (21, 22). This aligns with the treatment paradigm that emphasizes rapid intervention in the early phase of AIS. Additionally, recent findings by Zhao et al. have highlighted additional benefits of tirofiban, showing a significant reduction in the risk of early neurological deterioration (END) compared to aspirin in patients with AIS who did not receive reperfusion treatments (16). In our review, the analysis of this effect was not feasible as limited data were available. Larger studies exploring the role of tirofiban in preventing END in this specific cohort are needed.

Previous studies have suggested potential improvements in functional outcomes among AIS patients undergoing IVT or EVT with tirofiban (8, 9, 23). Furthermore, in a substantial trial involving 1,177 patients, tirofiban resulted in a higher likelihood of achieving excellent functional outcomes (defined as a mRS score of 0–1) at 90 days compared to aspirin for individuals with acute ischemic stroke lacking large or medium vessel occlusion (24). In contrast, the Safety of Tirofiban in Acute Ischemic Stroke (SaTIS) trial did not reveal improved functional outcomes of tirofiban over placebo in moderate stroke cases (25). Patients in these studies had the option of receiving reperfusion treatment with tirofiban. In this review, the pooled data indicated a notably increased rate of excellent functional outcomes at 90 days in the tirofiban-treated group. Further research is warranted to explore the effect of tirofiban in specific patient populations, including patients with large or medium vessel occlusion ineligible for EVT and those with stroke related to intracranial atherosclerotic disease (ICAD). ICAD is a prevalent cause of stroke in Asian and non-white populations, including Black and Hispanic groups (26). In case of ICAD-related stroke, up to half of patients encounter re-occlusion post-EVT, primarily due to preexisting atherosclerotic plaque rupture triggering platelet activation (27). The activated GP IIb/IIIa platelet receptors bind with fibrinogen, promoting platelet aggregation and thrombosis. Through targeted inhibition of GP IIb/IIIa, tirofiban can impede fibrinogen binding, thereby reducing the risk of subsequent thrombosis (28). In a recent meta-analysis, tirofiban had the potential to reduce re-occlusion rates in AIS patients treated with EVT, with particular efficacy noted in cases of ICAD-related stroke (29).

The safety of tirofiban in patients with AIS has been consistently reported in multiple studies. In a recent analysis, tirofiban was associated with a lower rate of sICH and mortality in patients with posterior circulation stroke undergoing EVT (29). In our review, a non-significant lower mortality rate at 90 days was noted in the tirofiban group. This is consistent with earlier reviews which indicated that tirofiban is potentially associated with reduced mortality among patients with AIS receiving IVT or EVT (23, 29, 30). The SaTIS trial has also reported a reduced mortality rate in the tirofiban group after a 5-month follow up (25). Although sICH definitions varied across included trials, potentially affecting the reported bleeding rates, our analysis observed a trend toward a lower rate of sICH rate in the tirofiban group. This could be attributed to aspirin’s pharmaceutical properties of non-selective and irreversible platelet aggregation (22). In contrast, tirofiban reversibly binds to glycoprotein IIb/IIIa receptors while possessing a short half-life, thereby normalizing bleeding time in about 3 h after its discontinuation (21). Our findings are consistent with previous reports from Zhou et al., which investigated the safety of tirofiban as a monotherapy and as a combination with IVT (31). The results from Zhou et al. analysis showed that neither group exhibited increased bleeding risk or mortality. Conversely, in the Efficacy and Safety of Tirofiban Compared with Aspirin in the Treatment of Acute Ischemic Stroke (RESCUE BT2) trial, the tirofiban group showed a slightly higher sICH rate than the aspirin group (1% vs. 0%), but the bleeding risk was low overall.

The low rate of intracranial bleeding found in our study can be attributed to several factors. Firstly, most of the studies included in our analysis primarily involved participants with minor to moderate stroke, as evidenced by median NIHSS scores ranging from 5 to 9, and a reduced risk of ICH can be expected in patients with minor baseline symptoms (32). On the contrary, individuals with acute minor strokes treated with reperfusion treatments might face an elevated risk of ICH with restricted treatment advantages, making IV tirofiban a potentially viable choice (33). Nevertheless, this study was not specifically designed to address this issue, and additional research is needed. Secondly, two of the included studies have excluded patients with cardioembolic stroke, and this population usually has a large infarction core and is at a higher risk of hemorrhagic transformation (34, 35). Overall, these findings collectively support the notion that tirofiban is safe in the early phase management of AIS, particularly for AIS cases where IVT or EVT cannot be given.

### 4.1 Limitations

There are several limitations that should be acknowledged. Firstly, most of the studies were conducted in China, with only one study from Italy (13–16). This can limit the generalizability of results to a broader population due to differences in baseline patients’ characteristics, stroke etiology, and vascular risk factors. Secondly, there were inconsistencies in the control group treatments and in the timing windows for tirofiban administration across the included trials. These variations may confound comparative outcomes and should be considered when interpreting the results. Thirdly, the inclusion of only four RCTs may hinder the ability to detect significant differences between the two groups. In addition, the small sample size of the included RCTs may have resulted in an overestimation of the beneficial treatment effects. Lastly, this analysis was a study-level aggregate meta-analysis rather than patient-level data. This limitation can restrict further analysis such as dedicated subgroup analyses and exploration of the role of potential confounders.

## 5 Conclusion

In conclusion, our meta-analysis showed that tirofiban holds promise for patients with AIS who did not undergo IVT or EVT therapy at the acute phase of stroke. Our results showed that tirofiban can enhance long-term functional outcomes without a corresponding increased intracranial bleeding risk. Further studies are warranted to explore tirofiban’s potential benefits further.

## Data Availability

Publicly available datasets were analyzed in this study. The primary data can be found in the original randomized controlled trials (references 13–16). Additional data are available upon request from the corresponding author. The search strategy and risk of bias assessment tables are provided in the Supplementary material.
