# Supplementary material for: Intravenous tirofiban in acute ischemic stroke patients not receiving reperfusion treatments: a systematic review and meta-analysis of randomized controlled trials
Source: Front Neurol. 2025 May 13;16:1552658. doi: 10.3389/fneur.2025.1552658 (PMC12108805; doi:10.3389/fneur.2025.1552658)
Supplement: Supplementary file 1 [file Table_1.docx]

Supplementary Material

**Supplemental Material**

**Table S1.** Search algorithms.

**Table S2.** Evaluation of study risk of bias using Risk of Bias 2 (RoB2) tool for randomized clinical trials.

**Table S1.** Search algorithms.

| **Database** | **Searches** | **Results** |
| --- | --- | --- |
| Embase | (((((((((('stroke') OR ('cerebrovascular accident'/exp)) OR ('ischemic stroke')) OR ('ischemic stroke'/exp)) OR ('cerebral infarction')) OR ('brain infarction'/exp)) OR ('cerebrovascular apoplexy')) OR ('cerebrovascular accident')) OR ('brain vascular accident')) OR ('cerebrovascular accident'/exp)) AND (('tirofiban') OR ('tirofiban'/exp)) | 1700 |
| CENTRAL | (((((((((("stroke"[All Fields]) OR ("stroke"[MeSH Terms])) OR ("ischemic stroke"[All Fields])) OR ("ischemic stroke"[MeSH Terms])) OR ("cerebral infarction"[All Fields])) OR ("cerebral infarction"[MeSH Terms])) OR ("cerebrovascular apoplexy"[All Fields])) OR ("cerebrovascular accident"[All Fields])) OR ("brain vascular accident"[All Fields])) OR (brain vascular accident[MeSH Terms])) AND (("tirofiban"[All Fields]) OR ("tirofiban"[MeSH Terms])) | 554 |
| WoS | ((((((ALL=(stroke)) OR ALL=(ischemic stroke)) OR ALL=(cerebral infarction)) OR ALL=(cerebrovascular apoplexy)) OR ALL=(cerebrovascular accident)) OR ALL=(brain vascular accident)) AND ALL=(tirofiban) | 465 |
| Medline | (((((((((("stroke"[All Fields]) OR ("stroke"[MeSH Terms])) OR ("ischemic stroke"[All Fields])) OR ("ischemic stroke"[MeSH Terms])) OR ("cerebral infarction"[All Fields])) OR ("cerebral infarction"[MeSH Terms])) OR ("cerebrovascular apoplexy"[All Fields])) OR ("cerebrovascular accident"[All Fields])) OR ("brain vascular accident"[All Fields])) OR (brain vascular accident[MeSH Terms])) AND (("tirofiban"[All Fields]) OR ("tirofiban"[MeSH Terms])) | 336 |

**Table S2.** Evaluation of study risk of bias using RoB2 tool for randomized clinical trials.

| **Study ID** | **Randomization**  **process** | **Deviation from intended intervention** | **Missing outcome data** | **Measurement of the outcome** | **Selection of the reported results** | **Overall** |
| --- | --- | --- | --- | --- | --- | --- |
| Torgano et al., 2010 [13] | Low | Low | Low | Low | Low | Low |
| Han et al., 2022 [14] | Low | Low | Low | Low | Low | Low |
| Yu et al., 2022 [15] | Low | Low | Low | Low | Low | Low |
| Zhao et al., 2024 [16] | Low | Low | Low | Low | Low | Low |
